# Exercise in Hypoxic Environments: An Overview of Systematic Reviews on Performance, Physiological Adaptation, and Clinical Implications

**DOI:** 10.3390/sports14040147

**Published:** 2026-04-10

**Authors:** Héctor Fuentes-Barría, Raúl Aguilera-Eguía, Miguel Alarcón-Rivera, Lisse Angarita-Davila, Eduardo Pena, Samia El Alam, Cherie Flores-Fernández

**Affiliations:** 1 Centro de Investigación en Medicina de Altura (CEIMA), Universidad Arturo Prat, Iquique 1110939, Chile; eduardopena@unap.cl (E.P.); selalam@unap.cl (S.E.A.); 2 Departamento de Salud Pública, Facultad de Medicina, Universidad Católica de la Santísima Concepción, Concepción 3349001, Chile; raguilerae@ucsc.cl; 3 Escuela de Ciencias del Deporte y Actividad Física, Facultad de Salud, Universidad Santo Tomas, Talca 3460000, Chile; mrivera3@santotomas.cl; 4 Escuela de Nutrición y Dietética, Facultad de Medicina, Universidad Andres Bello, Concepción 3349001, Chile; lisse.angarita@unab.cl; 5 Departamento de Gestión de la Información, Universidad Tecnológica Metropolitana, Santiago 7550000, Chile; cflores@utem.cl

**Keywords:** exercise, hypoxic, altitude sickness, athletic performance, adults

## Abstract

**Objectives:** This overview of systematic reviews aimed to synthesize and critically evaluate the current evidence on the effects of exercise performed under hypoxic or altitude conditions in adults, with particular attention to studies reporting altitude-related clinical outcomes. **Materials and Methods:** Following PRIOR and PRISMA guidelines, and with the protocol registered in PROSPERO CRD420261325746, a comprehensive search was conducted on 22 February 2026 across Medline/PubMed, Scopus, Web of Science, Epistemonikos, and Preprints.org, using the query “Exercise AND Hypoxic AND Altitude Sickness.” Because the search included “Altitude Sickness,” this review may miss some studies on performance or physiological adaptations under hypoxia. Eligibility was defined according to the PICOS framework, including only systematic reviews with or without meta-analyses in adults exposed to normobaric or hypobaric hypoxia. Methodological quality was assessed using AMSTAR 2. **Results:** A total of 137 records were identified (114 from databases and 23 through citation tracking), of which 28 systematic reviews met inclusion criteria. Nineteen included quantitative meta-analyses. Structured altitude training strategies—live high–train low (LHTL), live low–train high (LLTH), and live high–train high (LHTH)—were generally associated with improvements in maximal oxygen uptake and hematological parameters, particularly in trained and athletic populations. In contrast, acute hypoxic exposure was consistently associated with reduced exercise performance and increased susceptibility to altitude-related symptoms in unacclimatized individuals. Evidence regarding effects on body composition and metabolic outcomes was heterogeneous and inconsistent. According to AMSTAR 2, most meta-analyses presented critically low or low methodological quality. **Conclusions:** Exercise under hypoxic conditions may enhances aerobic and hematological adaptations in trained populations, whereas acute exposure tends to impair performance and entails clinical risks. However, given the restricted search strategy, substantial heterogeneity, lack of formal overlap quantification, and the predominance of low methodological quality reviews, these findings should be interpreted with caution. Evidence on metabolic benefits remains limited, highlighting the need for further high-quality systematic reviews and meta-analyses to clarify optimal hypoxic training protocols and outcomes.

## 1. Introduction

Regular physical exercise is a structured, planned, and repetitive form of physical activity performed with the objective of improving or maintaining physical fitness and health. It includes aerobics, resistance, interval, and combined training modalities prescribed according to frequency, intensity, time, and type [1,2]. From a physiological perspective, exercise requires precise coordination of cardiovascular, respiratory, hematological, and metabolic systems to ensure adequate oxygen delivery and energy production [3].

When performed under normoxic conditions, these mechanisms operate within relatively stable oxygen availability. However, environmental hypoxia—whether experienced at terrestrial altitude or simulated under normobaric (decrease in oxygen level) and hypobaric hypoxia (reduction in the partial pressure of inspired oxygen)—challenges oxygen transport and utilization pathways [4,5]. Altitude is defined as elevation above mean sea level and is commonly classified as moderate (1500–2500 m), high (≥2500–3500 m), very high (3500–5500 m), and extreme (>5500 m) [6]. The 2500 m threshold is particularly relevant, as it marks the level at which clinically meaningful arterial desaturation typically occurs in unacclimatized individuals at rest, increasing susceptibility to altitude-related conditions [6,7].

Environmental hypoxia elicits complex multisystem physiological adaptations affecting oxygen transport, metabolic regulation, and physical performance. Acute exposure is characterized by reductions in arterial oxygen saturation, increased ventilation and heart rate, alterations in cerebral oxygenation, and impaired exercise tolerance [3,8]. Over time, compensatory mechanisms may include stimulation of erythropoietin, increases in hemoglobin concentration, hematocrit, and red blood cell mass [9]. Over the past decades, hypoxic exposure combined with exercise has been widely implemented in both clinical and athletic contexts [10]. In sports science, altitude and hypoxic training strategies—such as live high–train low (LHTL), live low–train high (LLTH), and live high–train high (LHTH)—have been used to enhance erythropoietic responses and improve aerobic capacity [11].

In parallel, hypoxic exercise interventions have been explored as therapeutic approaches for overweight, obesity, and cardiometabolic disorders due to their potential to influence substrate utilization, insulin sensitivity, and body composition [12]. Conversely, acute hypoxic exposure during exercise may impair performance and increase susceptibility to altitude-related illnesses, including acute mountain sickness (AMS), high-altitude pulmonary edema (HAPE), and high-altitude cerebral edema (HACE) [7,13,14].

Despite the expanding body of literature, findings remain heterogeneous and, at times, contradictory. Some studies report significant improvements in hematological parameters and maximal oxygen uptake following structured altitude training, whereas others demonstrate minimal or no additional benefit of hypoxic exercise over normoxic conditions for anthropometric or metabolic outcomes in overweight or obese individuals [13,15,16,17]. Moreover, acute hypoxia has been consistently associated with reductions in overall exercise performance, particularly in untrained populations, highlighting the context-dependent nature of hypoxic adaptations. Variability in hypoxic dose (fraction of inspired oxygen, equivalent altitude, duration and frequency of exposure), training status, exercise modality, and population characteristics further complicates interpretation [13,15,16,17].

It is worth noting that, while numerous systematic reviews and meta-analyses have examined specific aspects of exercise under hypoxic conditions, there is still little literature that critically synthesizes the field [13]. Therefore, the aim of this overview of systematic reviews was to synthesize and critically evaluate the current evidence on the effects of exercise performed under environmental hypoxia on physiological, hematological, metabolic, and performance-related outcomes, with a particular focus on studies that also report altitude-related clinical outcomes, such as acute mountain sickness, HAPE, or HACE. This approach provides a consolidated and clinically relevant perspective on the role of hypoxic exercise in health optimization and performance enhancement, while acknowledging that not all performance-focused studies may have been captured.

## 2. Materials and Methods

### 2.1. Design

The protocol was developed following the recommendations outlined in the Preferred Reporting Items for Overviews of Reviews (PRIOR) and the guidelines of the Preferred Reporting Items for Systematic Reviews and Meta-Analyses (PRISMA) [18,19]; approval code: PROSPERO CRD420261325746.

Although the PROSPERO registration was submitted after the database search had been initiated, it was completed prior to screening, data extraction, and analysis. Therefore, while the registration was not strictly prospective, the protocol provides a pre-specified framework for study selection, eligibility criteria, and outcomes, ensuring transparency and methodological rigor throughout the review process.

At the time of registration in PROSPERO, the review team consisted of two researchers; other co-authors joined the project before the data selection and extraction stages, and their contributions are detailed in the “Author Contributions” section.

Therefore, the PROSPERO registration should not be interpreted as a strictly prospective methodological safeguard, but rather as a transparency mechanism that partially pre-specified key aspects of the review process.

### 2.2. Eligibility Criteria

Eligibility for study inclusion was determined using the P.I.C.O.S framework, which includes participants, intervention, comparison, outcome, and study design, with the following specifications:

- Participants: Adults (≥18 years), healthy, athletes, or individuals with metabolic conditions (e.g., overweight/obesity). Given the inclusion of diverse populations, heterogeneity across studies is expected and should be considered when interpreting pooled outcomes.
- Intervention: Exercise or physical training performed under environmental hypoxia (acute or chronic), including exercise testing in hypoxia, altitude training protocols (LHTL, LLTH, LHTH), or hypoxic exposure during exercise.
- Comparison: Exercise or training performed under normoxic conditions (sea level, FiO_2_ ~21%), standard exercise protocols without hypoxic exposure, or alternative hypoxic protocols for comparison within the same study.
- Outcome: Outcomes related to physical performance, aerobic capacity (VO_2_max, TTE, TT, sprint), erythrocyte mass and hematological parameters, peripheral oxygen saturation (SpO_2_), physiological responses to exercise, cardiometabolic factors, body composition, and/or prediction or development of high altitude-related illnesses (AMS, HAPE, HACE).
- Study design: Only systematic reviews with or without meta-analyses conducted in humans were included.

Editorials, primary studies, narrative reviews, and reviews that did not evaluate exercise under hypoxic or altitude conditions were excluded.

### 2.3. Data Sources and Search

A comprehensive electronic search was conducted on 22 February 2026 to identify relevant systematic reviews, in accordance with the protocol registered in PROSPERO. The following databases were consulted: Medline/PubMed, Scopus, Web of Science, Epistemonikos, and Preprints.org.

The search strategy combined controlled vocabulary (Medical Subject Headings [MeSH], when available) and free-text terms using Boolean operators, based on the registered query:

“Exercise AND Hypoxic AND Altitude Sickness”.

The syntax was adapted to each database as appropriate. No language restrictions were applied. Results were filtered by methodological design in each database as follows: “Systematic Review” in Medline/PubMed; “Review” in Scopus; “Review Article” in Web of Science and Epistemonikos. Additionally, the reference lists of included reviews were examined to identify further relevant studies.

It is important to note that this search strategy was intentionally restricted by the inclusion of the term ‘Altitude Sickness’, which shaped the scope of the evidence base toward studies reporting clinical altitude-related outcomes. While this approach ensured clinical relevance, it may have systematically excluded reviews focused exclusively on performance or physiological adaptations under hypoxia. Therefore, this should not be interpreted merely as a limitation, but as a structural constraint of the evidence synthesis that may have influenced the overall findings and their generalizability.

For Preprints.org, the search yielded zero results. While this may reflect a genuine absence of relevant reviews on the platform at the time, it should also be acknowledged as a potential limitation of the platform’s search functionality or the need to adapt search syntax specifically for preprint repositories.

### 2.4. Study Selection and Data Collection

Two reviewers independently screened the titles, abstracts, and full texts of all identified studies to assess eligibility. Any disagreements were resolved by a third reviewer, who acted as an arbitrator. Relevant data were extracted from each study, including first author, publication year, study population, interventions or comparisons, primary outcomes, and methodological characteristics. In cases of missing or unclear information, attempts were made to contact the corresponding author for clarification.

### 2.5. Methodological Quality Assessment

The methodological quality of the included meta-analyses was assessed using A Measurement Tool to Assess Systematic Reviews 2 (AMSTAR 2) [20], which comprises 16 domains designed to evaluate the rigor and reliability of systematic reviews. Each domain was rated as “Yes” (criterion met), “Partial Yes” (criterion partially met), or “No/Not applicable” (criterion not met or not applicable). The domains cover the following aspects: definition of the research question and inclusion criteria using PICO (#1); protocol registration (#2); explanation of study selection (#3); comprehensiveness of the literature search (#4); duplicate study selection (#5); duplicate data extraction (#6); listing of excluded studies with justification (#7); description of included studies (#8); assessment of risk of bias (#9); reporting of funding sources (#10); appropriateness of meta-analytic methods (#11); integration of risk of bias (#12); interpretation and discussion of risk of bias (#13); explanation of heterogeneity (#14); investigation of publication bias (#15); and declaration of conflicts of interest (#16).

The critical domains were identified as protocol registration (#2), comprehensive search (#4), listing of excluded studies (#7), risk of bias assessment (#9), appropriate meta-analytic methods (#11), consideration of risk of bias in the discussion (#13), and assessment of publication bias (#15). Following AMSTAR 2 guidance, overall confidence in each review was classified as High (no or one non-critical weakness), Moderate (multiple non-critical weaknesses but no critical flaws), Low (one critical flaw), or Critically Low (more than one critical flaw).

Assessments were independently performed by two researchers, with disagreements resolved by a third reviewer. Only reviews including meta-analyses were evaluated.

### 2.6. Assessment of Overlap Across Reviews

Given the nature of umbrella reviews, the potential overlap of primary studies across included reviews was considered. A full quantitative assessment of overlap (e.g., corrected covered area) was not conducted, as the inclusion of 28 systematic reviews encompassing multiple heterogeneous outcomes (e.g., aerobic performance, hematological adaptations, physiological responses, and metabolic variables) made the construction of a comprehensive citation matrix impractical and potentially misleading [21,22].

Instead, a targeted descriptive overlap matrix was developed based on outcome domains rather than individual primary studies [23]. This approach was selected to provide a structured and interpretable assessment of redundancy across reviews while accounting for the substantial heterogeneity in populations, interventions, and reported outcomes [21,23]. The matrix focused on the most frequently reported outcome domains, including aerobic capacity, hematological parameters, physiological responses to exercise, body composition, and sprint performance.

For each domain, the included systematic reviews were mapped and qualitatively classified according to the degree of overlap (high, moderate, or low), based on the recurrence of reviews addressing similar outcomes and the consistency of evidence synthesis across studies. This domain-based descriptive matrix allows for the identification of areas with a higher likelihood of overlapping evidence, while avoiding overinterpretation associated with constructing a full citation matrix in a highly heterogeneous evidence base [22,24].

This approach is consistent with methodological recommendations for umbrella reviews when a full overlap quantification is not feasible, particularly in cases involving multiple outcome domains and complex intervention heterogeneity [23,24].

Although this domain-based descriptive approach provides a structured overview of redundancy, it does not eliminate the risk of overlap across primary studies. Consequently, the possibility of double-counting evidence cannot be fully ruled out, which may affect the internal validity of the synthesis

## 3. Results

### 3.1. Study Selection

**Figure 1:**
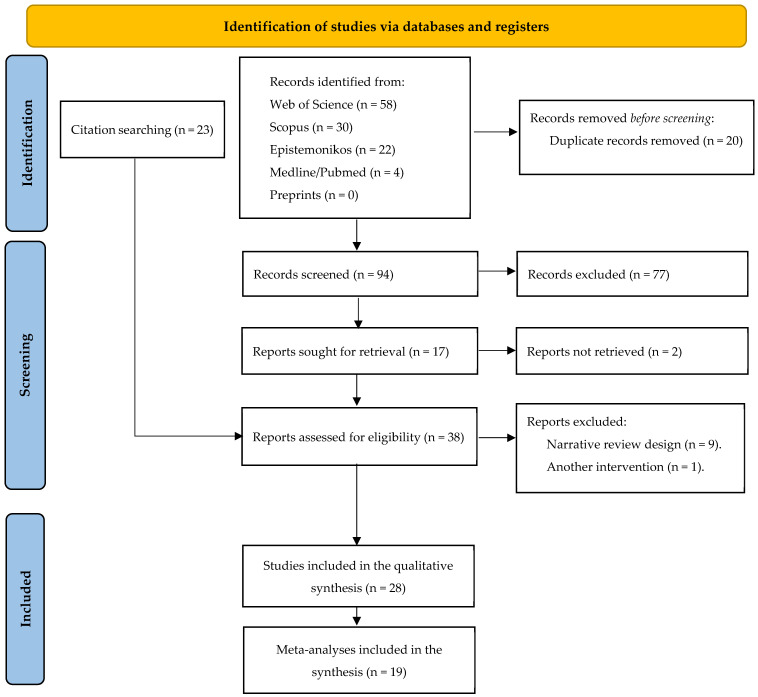
Study Selection Flow Diagram PRISMA 2020 [18]. This figure illustrates the process of studies via database and registers.

Figure 1 illustrates the search process conducted on 22 February 2026. A total of 114 records were identified through database searches, and no records were retrieved from preprints.org. All records were independently screened by two reviewers, with a third reviewer acting as an arbitrator to resolve disagreements.

After removal of 20 duplicate records, 94 studies remained for title and abstract screening. Of these, 77 were excluded, leaving 17 potentially eligible studies. Two of these could not be retrieved in full text [25,26]. The remaining 15 articles underwent full-text eligibility assessment [16,27,28,29,30,31,32,33,34,35,36,37,38,39,40]. Additionally, 23 further articles were identified through citation tracking [5,11,41,42,43,44,45,46,47,48,49,50,51,52,53,54,55,56,57,58,59,60,61], resulting in a total of 38 full-text articles assessed for eligibility.

Among these 38 full-text articles, 9 were excluded due to their narrative review design [27,28,29,30,31,32,33,34,35], and 1 was excluded for focusing on polymorphism-related interventions [36]. Ultimately, 28 articles were included in the qualitative synthesis [5,16,37,38,39,40,41,42,43,44,45,46,47,48,49,50,51,52,53,54,55,56,57,58,59,60,61], of which 19 contained quantitative meta-analyses [5,11,37,38,39,40,43,44,47,50,51,52,53,54,55,56,57,60,61].

### 3.2. Characteristics of Included Reviews

Table 1 summarizes the main characteristics of the systematic reviews included in this overview. The selected studies cover a wide range of populations, including healthy adults, elite athletes, and overweight or obese individuals. They evaluate diverse hypoxic exposure models, such as acute hypoxia (<24 h), simulated high altitude, and structured altitude training strategies, including LHTH, LHTL, and LLTH. The interventions range from acute hypoxic exercise testing protocols to chronic hypoxic training programs performed under normobaric or hypobaric conditions.

Reported outcomes include exercise performance parameters (e.g., time trial, time to exhaustion), aerobic capacity (VO_2_max), hematological adaptations (RBC, Hb, Hct, EPO), cardiometabolic markers (glucose, insulin, lipid profile), body composition variables, and physiological responses such as oxygen saturation (SpO_2_), ventilatory, and cardiac adaptations. Most studies were systematic reviews and meta-analyses [5,11,37,38,39,40,43,44,47,50,51,52,53,54,55,56,57,60,61], while one review synthesized evidence from cohort studies [16], reflecting methodological variability across the literature.

### 3.3. Aerobic Capacity and Physical Performance

Table 2 summarizes the effects of hypoxic or altitude-based training interventions on aerobic capacity and physical performance outcomes. Overall, most meta-analyses reported significant improvements in VO_2_max, although the magnitude of effect and heterogeneity varied considerably across studies.

Significant increases in VO_2_max were observed by Huang et al. [5], Park et al. [37], Lancaster et al. [43], Westmacott et al. [54], Fentaw et al. [57], and Chen et al. [61], supporting the effectiveness of structured hypoxic training strategies in enhancing aerobic capacity. However, heterogeneity ranged from moderate to high in several analyses, suggesting substantial variability in training protocols, participant characteristics, and hypoxic exposure doses. In contrast, Brocherie et al. [47] reported non-significant effects on VO_2_max, indicating that benefits may depend on intervention type or athletic population.

Beyond maximal oxygen uptake, additional performance-related variables showed mixed findings. Lancaster et al. [43] demonstrated reductions in maximal heart rate and improvements in peak exercise blood lactate concentration, whereas Guo et al. [38] found no significant effect on resting heart rate. Sprint- and repeated-sprint-related outcomes were also inconsistent: Ramos-Campo et al. [50] reported large improvements in lower-limb performance, and Précart et al. [56] observed significant improvements in sprint decrement score and maximal blood lactate, although repeated sprint ability indices were not consistently improved. Brocherie et al. [47] similarly found limited effects on repeated-sprint performance.

Notably, Deb et al. [39] demonstrated that acute hypoxic exposure exerted a clear ergolytic effect on time-trial and time-to-exhaustion performance, highlighting the important distinction between chronic hypoxic training adaptations and acute hypoxic responses.

Collectively, these findings indicate that structured hypoxic training generally enhances aerobic capacity, whereas outcomes related to sprint ability and short-duration efforts appear more protocol-, exposure-, and population-dependent.

Despite the consistent reporting of heterogeneity, the synthesis remains primarily descriptive and does not systematically stratify results by key moderators such as training status, hypoxic dose, or exposure modality. This limits the ability to draw more precise inferences from the aggregated evidence.

### 3.4. Hematological Adaptations

Table 3 summarizes the effects of hypoxic and altitude-based training interventions on hematological variables associated with oxygen transport capacity. Overall, most meta-analyses reported significant increases in hemoglobin (Hb), supporting the role of hypoxic exposure in stimulating erythropoietic adaptations. Significant improvements in Hb were observed by Huang et al. [5], Park et al. [37], Lancaster et al. [43], and Chen et al. [61], although heterogeneity ranged from low to very high depending on the review.

Park et al. [37] reported the most pronounced hematological responses, demonstrating significant increases in RBC, Hb, and Hct, but with very high heterogeneity (I^2^ > 90%), indicating substantial variability across included studies. Importantly, EPO levels were also significantly elevated in this review, supporting the mechanistic pathway of hypoxia-induced erythropoiesis. In contrast, Lancaster et al. [43] found no significant change in hematocrit despite improvements in hemoglobin, suggesting that plasma volume shifts or methodological differences may partly explain discrepancies across outcomes.

Collectively, these findings indicate that hypoxic training generally enhances hematological parameters related to oxygen-carrying capacity, particularly hemoglobin concentration and erythropoietic activity. However, the magnitude and consistency of these adaptations appear to depend on exposure dose, training model, and participant characteristics.

### 3.5. Body Composition and Metabolic Outcomes

Table 4 presents the effects of hypoxic and altitude-based training on body composition and metabolic variables. Overall, findings were more heterogeneous and less consistent than those observed for aerobic or hematological outcomes.

Guo et al. [38] reported no significant changes in BMI, waist-to-hip ratio, glycemia, or triglycerides, with generally low-to-moderate heterogeneity, suggesting limited metabolic benefits of hypoxic aerobic training in the analyzed populations. Similarly, Liu et al. [40] found a significant reduction in body fat ratio, whereas changes in body mass and BMI were not statistically significant. Most lipid profile variables and glycemic markers did not demonstrate consistent pooled effects and exhibited moderate-to-high heterogeneity, indicating substantial variability across intervention protocols and participant characteristics. Benavente et al. [44] also reported non-significant effects on hypertrophy, lean mass, and muscle thickness under moderate hypoxia, although small effect sizes suggested potential marginal benefits.

More favorable metabolic adaptations were observed by Ding et al. [60], who reported significant reductions in body mass, triglycerides, LDL cholesterol, and insulin resistance, all with negligible heterogeneity, suggesting more consistent findings within that analysis. Additionally, Ramos-Campo et al. [50] found a significant increase in muscle cross-sectional area, although lean mass changes were not statistically significant.

Taken together, these findings suggest that hypoxic training may confer modest improvements in body fat reduction and selected cardiometabolic risk markers—particularly in overweight or obese individuals—while overall effects on global body composition indices and lipid or glycemic parameters remain inconsistent and highly protocol-dependent.

Despite the consistent reporting of heterogeneity, the synthesis remains primarily descriptive and does not systematically stratify results by key moderators such as training status, hypoxic dose, or exposure modality. This limits the ability to draw more precise inferences from the aggregated evidence.

### 3.6. Comparative Effectiveness of Hypoxic Training Modalities

Table 5 provides a comparative overview of different hypoxic and altitude-based training modalities and their relative effectiveness on performance outcomes. Overall, findings indicate that both the type of hypoxic exposure and the exercise modality (aerobic vs. anaerobic) substantially influence training adaptations.

Deb et al. [39] demonstrated that acute hypoxic exposure induces marked performance decrements, particularly in untrained individuals and during short-duration efforts, highlighting the clear distinction between acute ergolytic effects and chronic adaptive responses. In contrast, Ramos-Campo et al. [50] reported small and non-significant differences between hypoxic and normoxic resistance training for lean mass, cross-sectional area, and lower-limb performance, suggesting that not all hypoxic interventions produce robust structural adaptations.

Network meta-analyses by Feng et al. [51] identified simulated and natural low-altitude “live high–train low” strategies as the most effective approaches for improving performance, whereas isolated hypoxic exposure and some intermittent hypoxic strategies showed more modest effects. When stratified by exercise modality, long-interval intermittent hypoxic training (l-IHT) consistently ranked highest for both aerobic and anaerobic performance, while certain combined interventions demonstrated limited or even negative effects, underscoring the importance of protocol design, intensity, and exposure structure.

Comparative analyses from Yu et al. [49] further indicated IHT and LHTL protocols generally outperform low-level normoxic training for both aerobic and anaerobic capacities, with moderate to large effect sizes across direct and indirect comparisons. Similarly, Hamlin et al. [55] and Bonetti et al. [11] reported consistent performance improvements with structured hypoxic high-intensity or live-high–train-low approaches. Chen et al. [61] additionally showed that alternating “Hi-Lo” models yielded greater performance gains than continuous “Hi-Hi” exposure, suggesting that intermittent altitude strategies may optimize adaptive responses.

Collectively, these findings emphasize that hypoxic training effectiveness is highly modality-dependent. Structured intermittent hypoxic interventions, particularly long-interval IHT and LHTL strategies, appear to provide the most robust and consistent performance benefits, whereas acute hypoxic exposure and poorly structured combined protocols may impair or fail to enhance performance.

### 3.7. Methodological Quality Assessment

Table 6 summarizes the methodological quality assessment of the included systematic reviews using AMSTAR 2, showing considerable variability in methodological rigor. Most reviews clearly defined their research questions using PICO components and reported the study selection process with PRISMA flow diagrams. However, protocol registration prior to conducting the review was uncommon. Comprehensive literature searches, including trial registries and grey literature, were frequently incomplete, and duplicate study selection and data extraction were inconsistently applied. Justification for excluded studies was often missing. Risk of bias assessment of primary studies was limited in several reviews, and meta-analytic methods, when applied, were sometimes inappropriate or insufficiently justified. Consideration of publication bias and integration of risk of bias into the interpretation of findings were generally inadequate.

Based on AMSTAR 2, the majority of included reviews were classified as critically low [5,11,37,38,39,40,41,42,43,44,45,46,47,48,49,50,51,52,54,55,56,60,61], while two reviews were rated as low quality [53,57]. These findings indicate that although systematic reviews provide useful evidence, their conclusions should be interpreted cautiously due to methodological limitations, particularly in critical domains such as protocol registration, comprehensive search strategy, risk of bias assessment, and publication bias evaluation.

### 3.8. Assessment of Overlap Across Reviews

A descriptive assessment of outcome coverage indicated that all outcomes were addressed in multiple reviews, although the extent of coverage varied across outcomes. Aerobic capacity was the most frequently reported outcome, included in 8 of the 19 reviews (42%), followed by body composition (5 reviews, 26%), hematological parameters (4 reviews, 21%), and sprint performance and physiological responses (3 reviews each, 16%). These findings highlight differences in the focus of reviews across the literature and point to areas with greater or more limited representation of evidence (Table 7).

## 4. Discussion

The studies included in this overview of systematic reviews evaluated the effects of diverse hypoxic training protocols on physiological, hematological, metabolic, and performance-related variables. As summarized in Table 1, the selected reviews encompassed heterogeneous populations, including athletes, healthy adults, and overweight/obese individuals, exposed to a broad range of interventions such as aerobic and resistance training, intermittent hypoxic exposure, and combined strategies (LLTH, LHTL, IHHE, RTH, RSH) under normobaric or hypobaric conditions. The duration of interventions ranged from 1 to 56 sessions, with simulated or terrestrial altitudes between 700 and 6875 m, or inspired oxygen fractions (FiO_2_) between 10–35% [5,11,16,37,38,39,40,41,42,43,44,45,46,47,48,49,50,51,52,53,54,55,56,57,58,59,60,61]. This variability highlights the wide methodological diversity within the field and partly explains the heterogeneity observed in pooled outcomes.

Although heterogeneity is consistently acknowledged across studies, the present overview does not fully disentangle its sources through structured stratified analyses (e.g., by training status, exposure type, or intervention characteristics). As a result, the synthesis primarily aggregates heterogeneous evidence, which limits interpretability and reduces the precision of the conclusions.

Quantitative findings indicate that both acute and chronic hypoxic exposure are associated with reductions in oxygen saturation and alterations in ventilatory and cardiovascular responses, occasionally accompanied by symptoms of acute mountain sickness (AMS). Nevertheless, in trained individuals, hypoxic training may enhance aerobic capacity (VO_2_max) [5,37,43,51,52,53,54,57,60,61] and hematological parameters such as hemoglobin, hematocrit, red blood cell count, and erythropoietin concentrations [5,37,43], although these effects should be interpreted cautiously rather than as definitive improvements product of the magnitude and consistency of these adaptations vary considerably across meta-analyses, with substantial heterogeneity reported in many pooled estimates. Therefore, while the direction of effect appears generally favorable in athletes, the strength of evidence supporting uniform benefits across protocols remains limited.

In addition, these adaptations are mediated by well-established molecular mechanisms, including activation of the hypoxia-inducible factor (HIF) pathway, which regulates erythropoietin production and angiogenesis, as well as mitochondrial biogenesis and metabolic enzyme modulation [62]. Repeated hypoxic exposure can stimulate increases in capillary density, oxidative enzyme activity, and mitochondrial content, enhancing oxygen delivery and utilization in skeletal muscle [63]. Such molecular responses are further influenced by training status, intensity, duration of exposure, and altitude level, emphasizing the mechanistic basis for observed heterogeneity across studies [64].

Regarding muscular adaptations, combined hypoxic training protocols have been associated with increases in muscle strength, hypertrophy, and aerobic capacity [44,47,50,51,52,53]. For instance, Ramos-Campo et al. [50] reported significant increases in muscle cross-sectional area (SMD = 0.70; 95% CI: 0.05–1.35; *p* = 0.04) and strength (SMD = 1.88; 95% CI: 1.20–2.56; *p* < 0.00001) in trained adults, while Bonetti et al. [57] observed improvements in aerobic performance with natural LHTL protocols (+4.0 ± 3.7%) and shorter artificial protocols (+2.6 ± 1.2%) in athletes. Differences in hypoxic dose, exercise modality, and participant characteristics limit direct comparability and may partially account for variability in reported effects. Notably, some effect size estimates from network meta-analyses appear implausibly large. For example, Yu et al. [53] reported indirect comparison SMDs of 6.94 (95% CI: 5.83–7.92) for IHT versus LLNT and 5.48 (95% CI: 4.28–6.56) for IHT versus IHE. Such extreme values are unlikely for physiological adaptations and likely reflect methodological artifacts inherent to indirect network comparisons, particularly when networks are sparse or when transitivity assumptions are violated. These estimates should be interpreted with caution, and readers should be aware that indirect comparisons in sparse networks may overestimate effect magnitudes.

Conversely, acute hypoxic exposure in untrained or healthy individuals may reduce exercise capacity and impair time-to-exhaustion and sprint performance [39]. This contrast underscores the importance of training status and adaptive capacity in modulating physiological responses to hypoxia. Similarly, effects on body composition and metabolic parameters appear inconsistent and strongly dependent on population characteristics and intervention duration [38,40,44,50], suggesting that metabolic adaptations may require longer or more precisely controlled exposure protocols.

From a practical standpoint, these mechanistic insights inform training prescription: for instance, higher altitudes or lower FiO_2_ may be required to elicit significant erythropoietic or mitochondrial adaptations, while session duration and cumulative exposure modulate the magnitude of these responses [5]. Training intensity should also be tailored, as excessive load under hypoxia in untrained individuals may increase fatigue or risk of overtraining [51]. Therefore, individualized programming based on athlete profile, fitness level, and specific adaptation goals is recommended [65].

The present overview aligns with previous narrative syntheses reporting that hypoxic exposure appears to enhance aerobic and hematological adaptations in trained individuals [27,28,29,30,31,32,33,34,35]. However, it extends prior work [13] by systematically evaluating methodological quality and synthesizing quantitative findings from 19 meta-analyses. Importantly, most included reviews were rated as critically low or low methodological quality according to AMSTAR 2. This creates a clear tension between the underlying evidence base and the strength of the conclusions. While positive effects on performance and physiological outcomes are frequently reported, the predominance of low-quality evidence substantially limits confidence in these findings. Therefore, any interpretation of beneficial effects should be considered tentative rather than definitive.

The heterogeneity observed across studies likely reflects differences in hypoxic stimulus (FiO_2_, simulated or terrestrial altitude), cumulative exposure duration, session frequency, exercise intensity, and participant characteristics. These factors not only influence physiological and performance outcomes but also complicate the translation of findings into standardized practical recommendations. Consequently, individualized hypoxic training approaches and careful differentiation of populations (athletic, healthy, clinical) remain essential when implementing such interventions [51,52,60,61].

Among the limitations of this overview, the generally low methodological quality of included reviews represents a primary constraint, limiting certainty in the synthesized conclusions. Additionally, although the search strategy was prospectively registered and adhered to predefined criteria, the inclusion of the term “Altitude Sickness” within the Boolean query may have restricted retrieval of reviews focused exclusively on performance or physiological adaptation without explicit reference to altitude-related pathology. While multiple databases were searched without language restrictions and citation tracking was performed to enhance coverage, the possibility of missed relevant reviews cannot be entirely excluded. Furthermore, although overlap of primary studies across systematic reviews is an inherent concern in umbrella reviews, a formal quantitative assessment (e.g., corrected covered area) was not conducted due to the substantial heterogeneity in outcomes, populations, and interventions included in this overview. Instead, a domain-based descriptive overlap approach was applied, which allowed the identification of patterns of redundancy across outcome categories. This assessment indicated a high degree of overlap for aerobic capacity outcomes, moderate overlap for hematological parameters and body composition, and low overlap for sprint performance and physiological responses. These findings suggest that redundancy is outcome-dependent, with greater convergence of evidence in aerobic outcomes and more heterogeneous evidence bases in other domains. Moreover, some pre-specified outcomes in the PROSPERO record, such as peripheral oxygen saturation (SpO_2_) responses and the incidence or prediction of altitude-related illnesses (AMS, HAPE, and HACE), were not systematically addressed in the included reviews. This may reflect limited reporting in the original reviews or insufficient extraction of these data in this overview, and should be considered when interpreting the results. In addition, the absence of a formal quantitative overlap assessment further limits confidence in the synthesis, as the potential duplication of primary studies across reviews may lead to an overrepresentation of certain findings. Finally, outcomes related to long-term metabolic health, body composition, and clinical endpoints remain underrepresented, limiting broader translational implications.

From a practical standpoint, structured hypoxic training strategies may enhance aerobic and hematological adaptations in trained and elite athletes if exposure is appropriately dosed and clinically monitored, whereas caution is warranted in untrained individuals, in whom acute hypoxic exposure may impair performance and increase physiological stress [13,66]. Thus, application should be context-specific and guided by individualized assessment.

It is important to note that, according to the PROSPERO record, this overview did not include a certainty assessment of the body of evidence using GRADE or an equivalent tool. This methodological choice was made because the primary aim of an overview of systematic reviews is to synthesize findings from previously published reviews rather than to directly evaluate the certainty of each primary outcome. Nevertheless, this limitation should be considered when interpreting the results, especially given that many of the included reviews were rated as low or critically low quality according to AMSTAR 2.

Future research should prioritize: (a) high-quality, preregistered systematic reviews and meta-analyses with rigorous risk-of-bias and publication bias assessments; (b) standardization of hypoxic exposure parameters (FiO_2_, equivalent altitude, duration, and cumulative dose) to enable meaningful comparisons; (c) investigation of long-term metabolic and clinical outcomes, particularly in overweight, obese, or clinical populations; and (d) stratified analyses by training status, sex, and age to enhance external validity and applicability.

## 5. Conclusions

Structured hypoxic training strategies (LHTL, LHTH, LLTH) appear to be associated with improvements in aerobic capacity and hematological parameters, particularly in trained populations. However, these findings are derived from a heterogeneous and predominantly low-quality evidence base. Acute hypoxic exposure during exercise appears to impair performance, especially in untrained individuals.

Given the restricted search strategy, substantial heterogeneity, absence of formal overlap quantification, and the predominance of critically low methodological quality reviews, the conclusions of this overview should be interpreted with caution. The current evidence does not allow for definitive statements regarding the magnitude or consistency of hypoxic training effects. Further high-quality systematic reviews and rigorously designed studies are required.

## Figures and Tables

**Table 1:** Characteristics of the selected studies (*n* = 28). Analysis of participants, intervention, altitude/doses of hypoxia, outcomes and design.

References	Participants	Intervention	Altitude/FiO_2_/SpO_2_	Outcomes	Design
Huang et al. [5]	349 athletes	LLTH (NA). (3–7 sessions/week for 9 days to 8 weeks,)	2500–5900 m	Aerobics performance	SR and MA
Bonetti et al. [11]	638 elite and subelite athletes	LLTH, LHTL, artificial long and brief LHTL, 5–47 days.	1800–6000 m	Aerobics performance	SR and MA
Georges et al. [16]	3558 healthy adults.	Different protocols of hypoxic exercise testing	5092 m	Ventilatory and cardiac responses, body composition and perceived sensations.	SR
Park et al. [37]	156 elite athletes	Aerobic exercise and intervals (4 h, 6 days/week for 4 weeks)	700–3000 m	Aerobics capacity	SR and MA
Guo et al. [38]	322 overweight/obese adults	Aerobic exercise, moderate intensity. (30 min/day, 5 days/week under hypoxia)	14–17.2% FiO_2_	Metabolic parameters and body composition	SR and MA
Deb et al. [39]	798 athletes	Normobaric and Hypobaric LLTH	5000–5700 m	Aerobics performance and capacity	SR and MA
Liu et al. [40]	1011 overweight/obese adults	Moderate-intensity aerobic exercise combined with resistance training (45–60 min, ≥4 days/week, ≤8 weeks).	1500–3000 m	Body composition and glycolipid metabolism	SR and MA
Behrendt et al. [41]	304 participants (34 athletes), older adults, CAD patients, young athletes with/without overtraining syndrome	Normobaric IHHE (3 sessions/week for 5 weeks).	10–35% FiO_2_	Exercise tolerance.	SR
Uzun et al. [42]	537 (11 athletes), male athletes.	IHHE, single 60-min session after heavy resistance exercise.	10–35% FiO_2_	Muscle damage and muscle recovery.	SR
Lancaster et al. [43]	160 athletes	Natural and artificial LHTL, brief intermittent hypoxia, 14–39 sessions.	1956–5500 m	Physical performance	SR and MA
Benavente et al. [44]	348 athletes	Normobaric LLTH (RTH). (2–4 sessions/week for 3–8 weeks).	12.7–16% FiO_2_	Muscle hypertrophy and strength development	SR and MA
Albertus-Cámara et al. [45]	202 athletes	Normobaric LLTH. (3–7 sessions/week for 1–13 weeks)	10–17% FiO_2_	Performance and hematological parameters	SR
Gamonales et al. [46]	527 trained and untrained adults	Normobaric LLTH. (2–4 sessions/week for 4–12 weeks).	NR	Strength and muscular hypertrophy	SR
Brocherie et al. [47]	202 athletes	Normobaric LLTH and Repeated sprint ability. (2.6 ± 0.6 sessions/week for 3.7 ± 1.3 weeks).	2900–3500 m	Aerobics and sprint performance	SR and MA
Seitz et al. [48]	259 trained/untrained adults	Normobaric LLTH (cycling and running). (2–5 sessions/week for 3–8 weeks).	2100–4500 m	Endurance capacity	SR
Zelenovic et al. [49]	347 athletes	LLTH (RSH). (5–18 sessions for 2–6 weeks).	13–14% FiO_2_	Performance	SR
Ramos-Campo et al. [50]	158 trained/untrained adults	LLTH (RTH). (2–3 sessions/week for 4–8 weeks).	12–16% FiO_2_	Performance strength and muscle growth.	SR&MA
Feng et al. [51]	1821 athletes	Hypobaric and normobaric LHTL, LHTH, IHT. 12–56 sessions.	690–5500 m	Performance	SR and MA
Feng et al. [52]	1040 trained adults	LLTH (RSH, l-IHT, ISH, IHE, HIIT). (2–7 sessions/week for 3–8 weeks).	1850–6875 m	Aerobics performance	SR and MA
Yu et al. [53]	1246 athletes and untrained adults	Normobaric and hypobaric LHTL, LLTH (IHE, IHT/IHIT, RSH, RTH). 10–56 sessions.	690–5500 m	Aerobics performance	SR and MA
Westmacott et al. [54]	194 athletes and untrained adults	HIIT under hypoxia. (2–13 weeks).	12.6–15.3% FiO_2_	Aerobics performance	SR and MA
Hamlin et al. [55]	187 athletes	LHTL, LHTLH, LLTH, IHT/IHE/RTH/RSH. (1–7 sessions/week for 1–5 weeks)	2500–4650 m	Aerobics performance.	SR and MA
Précart et al. [56]	199 trained adults	LLTH (RSN, RSH-VHL). (2–3 sessions/week for 2–6 weeks).	78.5–94.6 SpO_2_	Sprint performance	SR and MA
Fentaw et al. [57]	116 distance runners	HIIT under hypoxia.	2700–3000 m	Aerobics performance	SR and MA
Mclean et al. [58]	605 athletes	Hypobaric and Normobaric LLTH. (2 to 7 sessions/week for 1 to 7 weeks)	11.7–17% FiO_2_	Physical performance	SR
Yu et al. [59]	134 cyclist athletes	LLTH (1 to 4 sessions/week for 1 to 6 weeks)	1200–4000 m	Aerobics performance	SR
Ding et al. [60]	346 overweight/obese adults	Aerobic exercise training in hypoxia (20–90 min/session for 2–5 days/week during 3–32 weeks)	13–16.5% FiO_2_	Body composition and metabolic health	SR and MA
Chen et al. [61]	Athletes	Altitude training (Hi-Lo/Hi-Hi)	2000–2500 m	Performance and hematological parameters	SR and MA

CAD: Coronary Artery Disease; FiO_2_: Fraction of Inspired Oxygen; HIIT: High-Intensity Interval Training; Hi-Hi: Live High–Train High; Hi-Lo: Live High–Train Low; IHE: Intermittent Hypoxic Exposure; IHHE: Intermittent Hypoxic–Hyperoxic Exposure; IHIT: Intermittent Hypoxic Interval Training; IHT: Intermittent Hypoxic Training; ISH: Intermittent Sprint Training in Hypoxia; LLTH: Live Low–Train High; LHTL: Live High–Train Low; LHTLH: Live High–Train Low and High; LHTH: Live High–Train High; l-IHT: long Intermittent Hypoxic Training; MA: Meta-Analysis; NA: Not Available; NR: Not Reported; RSH: Repeated Sprint Training in Hypoxia; RSH-VHL: Repeated Sprint Training in Hypoxia—Very High Load; RSN: Repeated Sprint Training in Normoxia; RTH: Resistance Training in Hypoxia; SR: Systematic Review; SpO_2_: Peripheral Oxygen Saturation.

**Table 2:** Aerobic performance and physical performance.

Reference	Outcomes	Result
Huang et al. [5]	VO_2_max	WMD = 3.20 (95% CI: 1.33 to 5.08); I^2^ = 76.6%; *p* < 0.001
Park et al. [37]	VO_2_max	MD = 1.637 (95% CI: 0.381 to 2.894); *p* = 0.011; I^2^ = 89.3%
Guo et al. [38]	HR at rest	MD = 2.46 (95% CI: −2.88 to 7.80); I^2^ = 87%; *p* = 0.37
Deb et al. [39]	Time trial	−16.2% (95% CI: −22.9 to −9)
	Time to exhaustion	−44.5% (95% CI: −51.3 to −36.7)
	Sprint performance	−2.9% (95% CI: −16.5 to 12.8)
Lancaster et al. [43]	VO_2_max	WMD = 1.51 (95% CI: 0.44 to 2.58); I^2^ = 59%; *p* = 0.006.
	VO_2_max last 9.5 h	WMD = 3.45 (95% CI: 0.30 to 6.60). I^2^ = 34%; *p* = 0.03.
	HR max	WMD = − 1.77 (95% CI: −3.03 to −0.50). I^2^ = 45%; *p* = 0.006.
	PEBLC	WMD = −3.03 (95% CI: −4.57 to −1.49); I^2^ = 63%; *p* < 0.001.
Benavente et al. [44]	Strength	SMD = 0.13 (95% CI: −0.00 to 0.27); I^2^ = 13.2%; *p* = 0.11
Brocherie et al. [47]	VO_2_max	SMD = 0.18 (95% CI: −0.25 to 0.61); I^2^ = 0%; *p* = 0.41
	RSA	SMD = 0.46 (95% CI: −0.02 to 0.93); I^2^ = 6.19%; *p* = 0.05
	RSP	SMD = 0.31 (95% CI: −0.03 to 0.89); I^2^ = 6.19%; *p* = 0.30
Ramos-Campo et al. [50]	Lower Limb	SMD = 1.88 (95% CI: 1.20 to 2.56); I^2^ = 61%; *p* < 0.001
Westmacott et al. [54]	VO_2_max	SMD = 1.14 (95% CI: 0.56 to 1.72); I^2^ = 67%; *p* < 0.001
Précart et al. [56]	RSA best	SMD = 0.038 (95% CI: −0.252 to 0.328); I^2^ = 0%; *p* = 0.798
	RSA mean	SMD = 0.276 (95% CI: −0.018 to 0.570); I^2^ = 0%; *p* = 0.066
	Sprint decrement score	SMD = 0.603 (95% CI: 0.180 to 1.025); I^2^ = 40.87%; *p* = 0.005
	Max blood lactate	SMD = 0.611 (95% CI: 0.223 to 0.999); I^2^ = 0%; *p* = 0.002
Fentaw et al. [57]	VO_2_max	SMD = 0.68 (95% CI: 0.30 to 1.06); I^2^ = 0%; *p* < 0.001
Chen et al. [61]	VO_2_max	SMD = 0.67 (95% CI: 0.35 to 1.00); I^2^ = 29.8%; *p* < 0.001

CI: Confidence Interval; HR: Heart Rate; PEBLC: Peak Exercise Blood Lactate Concentration; RSP: Repeated Sprint Protocols; RSA: Repeated Sprint Ability; SMD: Standardised Mean Difference; VO_2_max: Maximal Oxygen Uptake or aerobic capacity; WMD: Weighted Mean Difference.

**Table 3:** Hematological variables.

Reference	Outcomes	Result
Huang et al. [5]	Hb	WMD = 0.25 (95% CI: 0.04 to 0.45); I^2^ = 59.4%; *p* = 0.002
Park et al. [37]	RBC	MD = 4.499 (95% CI: 2.469 to 6.529); *p* < 0.001; I^2^ = 93.4%
	Hb	MD = 5.447 (95% CI: 3.028 to 7.866); *p* < 0.001; I^2^ = 94.1%
	Hct	MD = 3.639 (95% CI: 1.687 to 5.591); *p* < 0.001; I^2^ = 93.3%
	EPO	MD = 0.711 (95% CI: 0.282 to 1.140); *p* = 0.001; I^2^ = 0%
Lancaster et al. [43]	Hb	WMD = 0.57 (95% CI: 0.38 to 0.75); I^2 ^ = 34%; *p* < 0.001.
	Hct	WMD = 0.60 (95% CI: −0.69 to 1.88); I^2^ = 0%; *p* = 0.36.
Chen et al. [61]	Hb	SMD = 0.50 (95% CI: 0.11 to 0.90); I^2^ = 0%; *p* = 0.013

Hb: Hemoglobin; Hct: Hematocrit; EPO: Erythropoietin; RBC: Red Blood Cell.

**Table 4:** Body composition and metabolic variables.

Reference	Outcomes	Result
Guo et al. [38]	BMI	MD = 0.29 (95% CI: −0.21 to 0.79); I^2^ = 22%; *p* = 0.25
	WHR	MD = 0.01 (95% CI: −0.01 to 0.03); I^2^ = 52%; *p* = 0.26
	Gly	MD = −0.02 (95% CI: −0.11 to 0.08); I^2^ = 57%; *p* = 0.75
	TG	MD = −5.75 (95% CI: −12.48 to 0.98); I^2^ = 34%; *p* = 0.09
Liu et al. [40]	Body mass	MD = −1.14 (95% CI: −2.34 to 0.07); I^2^ = 0%; *p* = 0.07
	Body fat ratio	MD = −1.16 (95% CI: −1.76 to −0.56); I^2^ = 42.9%; *p* < 0.001
	BMI	MD = −0.42 (95% CI −1.23 to 0.39); I^2^ = 69.55%; *p* = 0.31
	Total cholesterol	SMD = −0.04 (95% CI: −0.21 to 0.12); I^2^ = 39.4%; *p* = 0.59
	TG	SMD = 0.08 (95% CI: −0.20 to 0.35); I^2^ = 65.0%; *p* = 0.59
	LDL cholesterol	SMD = −0.23 (95% CI −0.65 to 0.19); I^2^ = 78.6%; *p* = 0.28
	HDL cholesterol	SMD = 0.17 (95% CI −0.26 to 0.59); I^2^ = 80.3%; *p* = 0.44
	Fasting blood glucose	SMD = 0.01 (95% CI: −0.16 to 0.19); I^2^ = 29.2%; *p* = 0.88
	Fasting blood insulin	SMD = 0.24 (95% CI: −0.30 to 0.79); I^2^ = 80.6%; *p* = 0.39
	Insulin Resistance	SMD = 0.04 (95% CI: −0.44 to 0.52); I^2^ = 71.5%; *p* = 0.86
Benavente et al. [44]	Hypertrophy	SMD = 0.21 (95% CI: −0.05 to 0.47); I^2^ = 16.3%; *p* = 0.06
	Lean mass	SMD = 0.02 (95% CI: −0.17 to 0.21); I^2^ = 0%; *p* = 0.87
	Muscle thickness	SMD = −0.06 (95% CI: −0.69 to 0.57); I^2^ = 77.4%; *p* = 0.87
Ramos-Campo et al. [50]	Lean Mass	SMD = 0.37 (95% CI: −0.13 to 0.88); I^2^ = 0%; *p* = 0.15
	CSA	SMD = 0.70 (95% CI: 0.05 to 1.35); I^2^ = 37%; *p* = 0.04
Ding et al. [60]	Body mass	MD = −0.90 (95% CI: −1.80 to −0.01); I^2^ = 0%; *p* = 0.05
	Fat body mass	MD = −1.22 (95% CI: −2.59 to 0.15); I^2^ = 0%; *p* = 0.08
	TG	MD = −10.78 (95% CI: −20.68 to −0.88); I^2^ = 0%; *p* = 0.03
	LDL cholesterol	MD = −3.74 (95% CI: −6.92 to −0.56); I^2^ = 0%; *p* = 0.02
	Insulin Resistance	MD = −0.22 (95% CI: −0.33 to −0.11); I^2^ = 0%; *p* < 0.001

BMI: Body Mass Index; CSA: Cross-Sectional Area; Gly: Glycemia; HDL: High Density Lipoprotein; LDL: Low Density Lipoprotein; TG: Triglycerides; WHR: Waist to Hip Ratio.

**Table 5:** Training modalities.

Reference	Outcomes	Result
Bonetti et al. [11]	HL_NAT/TLA	+4.0% (±3.7%)
	HL_SIM/TLA	+2.6% (±1.2%)
Deb et al. [39]	Trained individuals	−21.8% (95% CI: −31.2 to −11.1)
	Healthy/untrained	−29.5% (95% CI: −41.1 to −15.5)
	exercise <2 min	−5.6% (95% CI: −13.9 to 3.5)
	exercise >2 min	−4.7% (95% CI: −7.2 to −2.2)
	Overall effect	−17.1% (95% CI: −22.8 to −11)
Ramos-Campo et al. [50]	CSA vs. H	SMD = 0.24 (95% CI: −0.19 to 0.68); I^2^ = 37%; *p* = 0.27
	Lean Mass vs. H	SMD = −0.05 (95% CI: −0.56 to 0.46); I^2^ = 0%; *p* = 0.84
	Lower Limb vs. H	SMD = 0.20 (95% CI: −0.13 to 0.53); I^2^ = 61%; *p* = 0.23
Feng et al. [51]	HL_NAT/TLA	SMD = 1.04 (95% CrI: 0.47 to 1.61); *p*-score = 0.92
	HL_SIM/TLA	SMD = 0.91 (95% CrI: 0.44 to 1.38); *p*-score = 0.86
	HH	SMD = 0.61 (95% CrI: 0.26 to 0.96); *p*-score = 0.58
	HL_SIM/TSL	SMD = 0.56 (95% CrI: 0.17 to 0.95); *p*-score = 0.56
	HHL	SMD = 0.53 (95% CrI: −0.03 to 1.09); *p*-score = 0.53
	IHT	SMD = 0.36 (95% CrI: 0.10 to 0.62); *p*-score = 0.37
	IHE	SMD = 0.09 (95% CrI: −0.24 to 0.41); *p*-score = 0.13
Feng et al. [52]	Aerobic: l-IHT	SMD = 0.78 (95% CrI: 0.52 to 1.05); *p*-score = 1
	Aerobic: RSH	SMD = 0.30 (95% CrI: 0.10 to 0.50); *p*-score = 0.69
	Aerobic: s-IHT	SMD = 0.24 (95% CrI: −0.14 to 0.61); *p*-score = 0.57
	Aerobic: C + I	SMD = 0.18 (95% CrI: −0.13 to 0.50); *p*-score = 0.50
	Aerobic: IHE	SMD = 0.15 (95% CrI: −0.11 to 0.40); *p*-score = 0.44
	Aerobic: CHT	SMD = 0.10 (95% CrI: −0.31 to 0.50); *p*-score = 0.36
	Aerobic: ISH	SMD = 0.02 (95% CrI: −0.45 to 0.49); *p*-score = 0.28
	Anaerobic: l-IHT	SMD = 0.97 (95% CrI: 0.12 to 1.81); *p*-score = 0.95
	Anaerobic: SIH	SMD = 0.40 (95% CrI: 0.05 to 0.76); *p*-score = 0.71
	Anaerobic: RSH	SMD = 0.32 (95% CrI: 0.10 to 0.55); *p*-score = 0.62
	Anaerobic: s-IHT	SMD = 0.32 (95% CrI: 0.05 to 0.59); *p*-score = 0.61
	Anaerobic: IHE	SMD = 0.10 (95% CrI: −0.33 to 0.53); *p*-score = 0.37
	Anaerobic: C + I	SMD = −1.16 (95% CrI: −2.29 to −0.04); *p*-score = 0.01
Yu et al. [53]	Direct: IHE vs. LLNT	SMD = 0.57 (95% CI: 0.14 to 1.01)
	Indirect: IHE vs. LLNT	SMD = 1.45 (95% CI: 0.33 to 2.55)
	Direct: IHT vs. LLNT	SMD = 3.78 (95% CI: 2.48 to 5.09); I^2^ = 81.80%
	Indirect: IHT vs. LLNT	SMD = 6.94 (95% CI: 5.83 to 7.92)
	Direct: LHTL vs. LLTL	SMD = 0.71 (95% CI: 0.04 to 1.39)
	Indirect: LHTL vs. LLTL	SMD = 2.76 (95% CI: 1.27 to 4.20)
	Direct: IHE vs. LLTL	SMD = −0.95, 95% CI: −1.17 to −0.20)
	Indirect: IHE vs. LLTL	SMD = −2.50 (95% CI: −3.57 to −1.31)
	Direct: IHT vs. LLTL	SMD = 0.73 (95% CI: 0.41 to 1.05)
	Indirect: IHT vs. LLTL	SMD = 2.99 (95% CI: 2.15 to 3.82)
	Direct: IHT vs. IHE	SMD = 3.09 (95% CI: 2.05 to 4.13)
	Indirect: IHT vs. IHE	SMD = 5.48 (95% CI: 4.28 to 6.56)
	RSH vs. LLNT	SMD = 0.37 (95% CI: −0.05 to 0.78)
	LHTH vs. LLTL	SMD = 0.40 (95% CI: −0.05 to 0.86)
	RSH vs. LLTL	SMD = 0.36 (95% CI: −0.11 to 0.82)
Hamlin et al. [55]	HIIT Intermittent	SEst = 0.78 ± 0.76 (90% CI)
	Live high	SEst = 0.78 ± 0.74 (90% CI)
Chen et al. [61]	Hi-Lo	SMD = 0.79 (95% CI: 0.33 to 1.26); I^2^ = 48.7%; *p* = 0.001
	Hi-Hi	SMD = 0.52 (95% CI: 0.02 to 1.00); I^2^ = 0%; *p* = 0.041

C + I: Combined Continuous + Intermittent; CHT: Continuous Hypoxic Training; CSA: Cross-Sectional Area; HIIT: High-Intensity Interval Training; HHL: Hypoxia High/Low (mixed protocol); HH: Hypobaric Hypoxia; H: Hypoxia; HL_NAT/TLA: Natural Hypoxia, Live High Train Low Altitude; HL_SIM/TLA: Simulated Hypoxia, Live High Train Low Altitude; HL_SIM/TSL: Simulated Hypoxia, Train Slow/Low; Hi-Hi: Live High, Train High; Hi-Lo: Live High, Train Low; IHE: Intermittent Hypoxic Exposure; IHT: Intermittent Hypoxic Training; ISH: Intermittent Sprint in Hypoxia; l-IHT: Long Intermittent Hypoxic Training; LLNT: Live Low, No Training; LLTL: Live Low, Train Low; LHTH: Live High, Train High; LHTL: Live High, Train Low; RSH: Repeated Sprint in Hypoxia; SIH: Sprint Interval Hypoxia; s-IHT: Short Intermittent Hypoxic Training.

**Table 6:** Methodological quality assessment of the included studies.

References	N ° Items																Quality
	1	2	3	4	5	6	7	8	9	10	11	12	13	14	15	16	
Huang et al. [5]	Y	Y	N	PY	Y	Y	N	Y	Y	N	Y	N	N	Y	Y	Y	Critically Low
Bonetti et al. [11]	Y	N	Y	PY	N	N	Y	Y	PY	N	Y	Y	Y	Y	N	Y	Critically Low
Park et al. [37]	Y	N	Y	PY	N	N	PY	Y	N	N	Y	N	N	Y	N	Y	Critically Low
Guo et al. [38]	Y	Y	Y	PY	Y	Y	PY	Y	Y	N	Y	N	Y	Y	Y	Y	Critically Low
Deb et al. [39]	Y	N	Y	PY	Y	Y	N	Y	PY	N	Y	N	N	Y	N	Y	Critically Low
Liu et al. [40]	Y	Y	Y	PY	N	N	N	Y	Y	N	Y	N	N	N	Y	Y	Critically Low
Lancaster et al. [43]	Y	N	Y	N	Y	N	Y	Y	PY	N	Y	N	Y	Y	Y	Y	Critically Low
Benavente et al. [44]	Y	N	Y	Y	Y	Y	PY	Y	Y	N	Y	Y	Y	Y	Y	Y	Critically Low
Brocherie et al. [47]	Y	PY	Y	Y	N	N	N	Y	PY	N	Y	N	N	Y	Y	Y	Critically Low
Ramos-Campo et al. [50]	Y	N	Y	PY	Y	Y	Y	Y	Y	N	Y	Y	Y	Y	N	Y	Critically Low
Feng et al. [51]	Y	Y	Y	PY	Y	Y	PY	Y	Y	N	Y	Y	Y	Y	Y	Y	Critically Low
Feng et al. [52]	Y	Y	Y	PY	Y	N	PY	Y	Y	N	Y	Y	Y	Y	Y	Y	Critically Low
Yu et al. [53]	Y	Y	Y	Y	Y	Y	Y	Y	Y	N	Y	Y	Y	Y	N	Y	Low
Westmacott et al. [54]	Y	N	Y	PY	Y	Y	Y	Y	PY	N	Y	Y	Y	Y	Y	Y	Critically Low
Hamlin et al. [55]	Y	N	Y	PY	N	N	N	Y	N	N	Y	N	N	Y	N	Y	Critically Low
Précart et al. [56]	Y	N	Y	Y	Y	Y	N	Y	Y	N	Y	Y	Y	Y	Y	Y	Critically Low
Fentaw et al. [57]	Y	Y	Y	Y	Y	Y	PY	Y	Y	N	Y	Y	Y	Y	Y	Y	Low
Ding et al. [60]	Y	Y	Y	Y	Y	Y	N	Y	Y	N	Y	N	N	Y	N	Y	Critically Low
Chen et al. [61]	Y	N	Y	Y	Y	Y	N	Y	Y	N	Y	N	Y	Y	Y	Y	Critically Low

N: No/Not applicable; PY: Partial Yes; Y: Yes.

**Table 7:** Assessment of overlap across reviews.

Outcome Domain	Number of Reviews Included	Total Reviews	% of Total Reviews	Coverage Classification
Aerobic capacity	[5,33,39,43,47,50,53,57]	19	42	High
Sprint performance	[39,47,56]	19	16	Low
Hematological parameters	[5,37,43,61]	19	21	Moderate
Physiological responses	[39,43,56]	19	16	Low
Body composition	[38,40,44,50,60]	19	26	Moderate

High: >40% of Total Reviews, Moderate: 20–40% of Total Reviews, Low: <20% of Total Reviews.

## Data Availability

No new data were created or analyzed in this study. Data sharing is not applicable to this article.

## Abbreviations

The following abbreviations are used in this manuscript:

AMS	Acute Mountain Sickness
BMI	Body Mass Index
CAD	Coronary Artery Disease
CSA	Cross-Sectional Area
EPO	Erythropoietin
FiO_2_	Fraction of Inspired Oxygen
Gly	Glycemia
HACE	High altitude Cerebral Edema
HAPE	High Altitude Pulmonary Edema
Hb	Hemoglobin
Hct	Hematocrit
HH	Hypobaric Hypoxia
HL	Hypoxic Living
HHL	Hypoxia High/Low (mixed protocol)
HDL	High Density Lipoprotein
Hi-Hi	Live High, Train High
Hi-Lo	Live High Train Low
HIIT	High-Intensity Interval training conducted under Hypoxic conditions
HOMA-IR	Homeostatic Model Assessment of Insulin Resistance
HR	Heart Rate
IHE	Intermittent Hypoxic Exposure performed Passively
IHHE	Intermittent Hypoxia–Hyperoxia Exposure
IHT	Intermittent Hypoxic Training
ISH	Interval Sprint Training in Hypoxia
l-IHT	Long-duration Intermittent Hypoxic Training
LDL	Low Density Lipoprotein
LLTH	Live Low-Train High
MA	Meta-Analysis
PEBLC	Peak Exercise Blood Lactate Concentration
RBC	Red Blood Cell
RSH	Repeated Sprint Training in Hypoxia
RSH-VHL	Repeated Sprint training in Hypoxia induced by Voluntary Hypoventilation at Low Lung Volume
RSN	Repeated Sprint training in Normoxia
RTH	Resistance Training in Hypoxia
SMD	Standardised Mean Difference
SpO_2_	Peripheral Oxygen Saturation
SR	Systematic Review
TG	Triglycerides
VO_2_max	Maximal Oxygen Uptake or aerobic capacity
WHR	Waist to Hip Ratio
WMD	Weighted Mean Difference
